# TACE-HAlC combined with Donafenib and immune checkpoint inhibitors for BCLC stage C HCC patients (THEME study): a retrospective IPTW-adjusted cohort study

**DOI:** 10.3389/fimmu.2025.1669856

**Published:** 2025-11-11

**Authors:** Linan Yin, Weihang Li, Wencheng Shao, Xunbo Hou, Bowen Liu, Xuesong Liu, Ruibao Liu, Peng Huang

**Affiliations:** 1 Department of Interventional Radiology, Harbin Medical University Cancer Hospital, Harbin, China; 2 Department of Radiation Physics, Harbin Medical University Cancer Hospital, Harbin, China; 3 Department of Gastroenterology, Harbin Medical University Cancer Hospital, Harbin, China

**Keywords:** hepatocellular carcinoma, Donafenib, transarterial chemoembolization, hepatic arterial infusion chemotherapy, immune checkpoint inhibitors

## Abstract

**Background:**

Treatment options for Barcelona Clinic Liver Cancer (BCLC) Stage C hepatocellular carcinoma (HCC) remain limited, with targeted therapy combined with immune checkpoint inhibitors (ICIs) serving as the standard first-line treatment. This study investigates whether a combination of transarterial chemoembolization (TACE), hepatic arterial infusion chemotherapy (HAIC), donafenib, and ICIs (quadruple therapy) provides superior survival benefits compared to the targeted therapy combined with ICIs (targeted-immunotherapy).

**Methods:**

We conducted a retrospective analysis of patients with BCLC stage C HCC who received quadruple therapy or targeted-immunotherapy at the Harbin Medical University Cancer Hospital between September 2019 and October 2024The primary outcome was overall survival (OS). Secondary outcomes included progression-free survival (PFS), objective response rate (ORR), Disease Control Rate (DCR) and safety. To minimize baseline imbalances between the groups, we applied stabilized inverse probability of treatment weighting (sIPTW) methods.

**Results:**

A total of 195 patients were included in the study, of whom 125 were assigned to the quadruple therapy group and 70 to the targeted-immunotherapy Group. After applying sIPTW to balance the baseline characteristics between the two groups, patients in the quadruple therapy group demonstrated a significantly higher median OS (mOS) compared with the targeted-immunotherapy group (29.4 months [95% CI: 23.9-NA] vs 18.0 months [14.7-31.8]; P = 0.041). Additionally, the median PFS (mPFS) assessed by the modified Response Evaluation Criteria in Solid Tumors (mRECIST) was longer in the quadruple therapy group(16.4 months [95% CI: 12.7-NA] vs 10.0 months [3.32-31.8]; P = 0.012). Under the mRECIST criteria, quadruple therapy group demonstrated superior)ORR (68.4% vs 28.%, P = 0.001) and DCR (92.3% vs 63.1%, P < 0.001). The incidence of any adverse events (AE) in the quadruple therapy group was 95.2%, compared with 97.1% in the targeted-immunotherapy group. Among these the incidence of grade ≥3 AE was 40.8% in the quadruple therapy group and 38.6% in the targeted-immunotherapy group.

**Conclusions:**

Compared with targeted-immunotherapy group, patients with BCLC stage C HCC treated with TACE-HAIC combined with donafenib and ICIs demonstrated superior efficacy and acceptable safety.

## 1 Introduction

Hepatocellular carcinoma is the sixth most common malignancy globally and the third leading cause of cancer-related mortality, with an estimated annual incidence exceeding 800,000 new cases (1). This malignancy, predominantly arising in the context of chronic liver diseases such as hepatitis B, hepatitis C, or cirrhosis, poses a significant global health challenge (2). The BCLC staging system is a globally recognized framework widely employed for stratifying HCC patients and guiding therapeutic decisions. Within this system, BCLC stage C represents advanced liver cancer characterized by major vascular invasion, extrahepatic spread, or cancer-related symptoms. At this stage, curative treatment options such as surgery, liver transplantation, and ablation are no longer considered viable alternatives (3). For patients with BCLC Stage C HCC, systemic therapies have historically been the mainstay of treatment. The appearance of sorafenib, a multi-target tyrosine kinase inhibitor (TKI), marked a pivotal advancement by demonstrating a survival benefit in this population (4). More recently, the integration of TKIs with immune checkpoint inhibitors (ICIs), exemplified by regimens such as atezolizumab plus bevacizumab (5), has outperformed sorafenib alone, establishing a new standard of care for first-line treatment (6). Despite systemic regimens extending the mOS in uHCC to 22.1–24 months, patients with BCLC stage C still require more potent strategies to further improve clinical outcomes (6, 7).

Locoregional therapies, such as TACE and HAIC, have traditionally been utilized in intermediate-stage HCC (BCLC Stage B). TACE involves the targeted delivery of chemotherapeutic agents via the hepatic artery, followed by embolization to induce tumor necrosis (8), while HAIC employs continuous intra-arterial infusion to achieve elevated local drug concentrations with potentially reduced systemic toxicity (9). Emerging evidence suggests that combining these locoregional approaches with systemic therapies may enhance tumor control and prolong survival in advanced HCC (10, 11). Concurrently, previous studies have indicated that the combined application of TACE and HAIC—two distinct locoregional therapies—may further enhance therapeutic efficacy in patients with unresectable hepatocellular carcinoma (12, 13).Donafenib, a novel TKI structurally akin to sorafenib, has demonstrated better efficacy and an improved safety profile in phase III trials, positioning it as a promising alternative for advanced HCC management (14). When paired with ICIs, which bolster anti-tumor immunity by inhibiting immunosuppressive pathways such as PD-1/L1 or CTLA-4, this combination may yield synergistic effects.

However, no potent combination regimen has been reported in BCLC Stage C HCC patients. In this study, the efficacy and safety of combination therapy TACE-HAIC combined with Donafenib and ICIs were compared with those of targeted therapy plus ICIs in the treatment of HCC BCLC Stage C patients.

## 2 Materials and methods

We retrospectively reviewed data of BCLC Stage C HCC patients in Harbin Medical University Cancer Hospital from 2019 to 2024. This study was approved by the Institutional Review Board of Harbin Medical University Cancer Hospital and was performed in accordance with Declaration of Helsinki of 1975, as revised in 1983. The Ethics Committee of Harbin Medical University Cancer Hospital approved this study (approval number: 2024-428-IIT). The need for informed consent was waived by the institutional review board due to the retrospective nature of this study.

### 2.1 Patient population and data collection

Patients were selected based on specific inclusion criteria: (a) diagnosis of BCLC stage C HCC confirmed by histologic or cytologic analysis or clinical feature according to the American Association for the Study of Liver Diseases (AASLD) guideline (15) (b)Eastern Cooperative Oncology Group(ECOG) Performance Status ≤ 2; (c) Child-Pugh Class A or B);(d)age≥18; (e) patients received either targeted therapy (TKIs or bevacizumab) plus ICIs or a combination of TACE, HAIC, Donafenib, and ICIs as first-line therapy; (f) at least one measurable lesion within the liver; (g) availability of complete medical records with treatment and follow-up details. The exclusion criteria included: (a) have received previous systemic treatment; (b) uncorrectable abnormalities in liver and renal function, as well as coagulation disorders; (c) refractory heart and lung insufficiency; (d) presence of definitive contraindications to the use of targeted therapies and immune checkpoint inhibitors; (e) life expectancy less than 3 months.

### 2.2 Treatment protocol and assessment of response

#### 2.2.1 TACE

In this study, TACE was performed prior to HAIC in all cases, utilizing a femoral arterial access route. A standard 5F arterial sheath was placed in the femoral artery, through which a 4F hepatic artery catheter and a 2.1-2.5F microcatheter were used for comprehensive angiographic assessment of the celiac trunk and tumor-feeding arteries, such as the superior mesenteric artery and diaphragmatic arteries. Superselective catheterization techniques were employed for embolization. Depending on the tumor size and blood flow velocity, a mixture of iodized oil (5-20mL) and doxorubicin hydrochloride (20-60mg) was prepared into an emulsion, which was infused until a reduction in tumor vascular flow was achieved. In TACE, particulate embolic agents are generally not employed unless the tumor-feeding arterial flow remains rapid after lipiodol emulsion embolization or when the tumor is concurrently supplied by multiple feeding arteries.

#### 2.2.3 HAIC

Following TACE, a microcatheter is placed in the main trunk or primary branches of the tumor-feeding arteries, to which an external arterial chemotherapy pump is connected. The chemotherapy regimen employed is a modified FOLFOX4 protocol (14). The specific regimen consists of: intravenous infusion of oxaliplatin at 85mg/m^2^ over 2–3 hours via the arterial pump, followed by calcium folinate at 400mg/m^2^ infused over 1 hour, and bolus injection of fluorouracil (400mg/m^2^) into the artery. Subsequently, a continuous arterial infusion of fluorouracil at 2400mg/m^2^ is administered over 46 hours. Repeated TACE-HAIC was performed at intervals of 4–5 weeks. Depending on the patient’s clinical status, laboratory results, and cumulative treatment cycles, the chemotherapy dose will be dynamically adjusted, with a planned reduction in dosage after two cycles.

### 2.3 Systemic therapy

The targeted therapeutic agents employed in this study and their respective dosing regimens are as follows: Lenvatinib, 8 mg daily (for body weight ≤ 60kg) or 12mg daily (for body weight >60kg), administered orally; Donafenib, 0.2g twice daily, administered orally; Bevacizumab or bevacizumab analogs, 15mg/kg every 21 days, administered intravenously. The PD-1 inhibitors used in this study and their dosing regimens include: Camrelizumab, 200mg every 21 days, administered intravenously; Sintilimab, 200mg every 21 days, administered intravenously; Tislelizumab, 200mg every 21 days, administered intravenously; Toripalimab, 240mg every 21 days, administered intravenously; Pembrolizumab, 200mg every 21 days, administered intravenously; Atezolizumab, 1200mg every 21 days, administered intravenously. Targeted agents and immunotherapy are typically initiated within 3 to 7 days following interventional treatment. In the event of clinically significant adverse effects associated with systemic therapy, dose reduction or temporary discontinuation is considered, accompanied by appropriate symptomatic management. If adverse events of similar severity recur upon resumption of therapy, an alternative systemic treatment regimen should be instituted. During the interventional period, from the first 3 days to the last 3 days, systemic therapy was temporarily suspended.

### 2.4 Assessment of response and safety

Every 2–3 treatment cycles, a routine follow-up was conducted for all patients, which included liver contrast-enhanced CT or MRI, electrocardiogram, laboratory tests encompassing a biochemical panel, complete blood count, coagulation profile, alpha-fetoprotein (AFP), thyroid function tests, as well as clinical symptoms and signs. The mRECIST criteria were employed for evaluating the efficacy in participants, determining tumor response and progression. Tumor response categories post-treatment included Complete Response (CR), Partial Response (PR), Stable Disease (SD), or Progressive Disease (PD). The ORR was defined as the sum of CR and PR, while the DCR was defined as the sum of CR, PR, and SD. Treatment-related adverse events (TRAEs) were assessed according to the Common Terminology Criteria for Adverse Events (CTCAE) version 5.0 (16).

### 2.5 Statistical analysis

The study’s methodology involved a retrospective review of patient records, with statistical analyses tailored to each outcome and performed using Python programming under Anaconda 3 environment. In detail, the survival module was applied to conduct Kaplan-Meier survival analysis, and the scipy.stats module was used for statistical tests such as the log-rank test and chi-squared test. Baseline characteristics were summarized with descriptive statistics, such as means and standard deviations for continuous variables and frequencies and percentages for categorical ones.

To further address potential confounding due to baseline differences between treatment groups, Stabilized Inverse Probability of Treatment Weighting (sIPTW) was employed. For survival outcomes, Kaplan-Meier curves were employed to visualize OS and PFS, while the log-rank test facilitated comparisons of survival distributions between the targeted-immunotherapy group and quadruple therapy group, both in unweighted and IPTW-weighted analyses (16). Additionally, to adjust for potential confounding variables, a Cox proportional hazards model was employed to assess the independent effect of the treatment group on OS and PFS, adjusting for relevant baseline characteristics and supplemented by IPTW-weighted estimates. Response rates, specifically ORR and DCR, were analyzed by comparing proportions between groups using the chi-squared test or Fisher’s exact test, selected based on sample size and expected frequencies, with IPTW applied to adjust for baseline imbalances (17). Furthermore, logistic regression models, enhanced by IPTW weighting, were used to evaluate the association between the treatment group and ORR as well as DCR, controlling for the same set of covariates. Safety profiles were evaluated by summarizing adverse events with descriptive statistics, and where sufficient data allowed, statistical comparisons between groups were conducted to assess differences in adverse event incidence, with IPTW adjustments applied as appropriate (16, 18). A p-value threshold of less than 0.05 was set for statistical significance across all analyses (19).

## 3 Results

### 3.1 Patient characteristics

A total of 195 patients were enrolled in this study, with 125 receiving a combination of TACE-HAIC combined with Donafenib and ICIs, while 70 patients underwent targeted therapy plus immunotherapy. The follow-up durations for quadruple therapy group and targeted-immunotherapy group were 26.64 months and 53.32 months, respectively. Prior to sIPTW, baseline data analysis revealed that the two groups had comparable mean ages. Both groups were predominantly male, with similar proportions of patients with viral hepatitis, as well as the Child-Pugh scores, AFP levels, and tumor counts, indicating similar general health and liver function status between the groups. However, significant differences were noted in tumor-related characteristics: quadruple therapy group had a larger average tumor diameter (100.85 mm, SD 45.30 vs. 91.54 mm, SD 36.84), a higher proportion of portal vein tumor thrombosis (PVTT), while the Targeted-Immunotherapy Group exhibited a higher incidence of extrahepatic metastasis. (**Table 1**)

**Table 1:** Baseline characteristics of the patients.

Variable		Before IPTW				sIPTW			
		A(N = 70)	B(N = 125)	P	SWD	A(N = 71)	B(N = 122)	P	SWD
Age (mean (SD))		56.86 (9.45)	56.10 (9.45)	0.594	0.080	56.63 (8.58)	56.86 (9.08)	0.879	0.026
Sex (%)	Male	58 (82.9)	100 (80.0)	0.766	0.074	63.6 (89.1)	101.3 (83.3)	0.256	0.169
	Female	12 (17.1)	25 (20.0)			7.8 (10.9)	20.3 (16.7)		
ECOG (%)	0	36 (51.4)	105 (84.0)	**<0.001**	**0.743**	51.9 (72.7)	88.5 (72.8)	0.995	0.001
	1	34 (48.6)	20 (16.0)			19.5 (27.3)	33.1 (27.2)		
Child–pugh(%)	A	57 (81.4)	103 (82.4)	1.000	0.025	55.8 (78.1)	97.1 (79.8)	0.858	0.043
	B	13 (18.6)	22 (17.6)			15.6 (21.9)	24.5 (20.2)		
BCLC (%)	C	70 (100.0)	125 (100.0)	NA	<0.001	71.4 (100.0)	121.6 (100.0)	NA	<0.001
CNLC (%)	IIIa	29 (41.4)	106 (84.8)	**<0.001**	**1.006**	49.7 (69.6)	98.4 (81.0)	0.124	0.266
	IIIb	41 (58.6)	19 (15.2)			21.7 (30.4)	23.2 (19.0)		
Etiology (%)	HBV	60 (85.7)	92 (73.6)	**0.102**	**0.339**	52.0 (72.8)	95.0 (78.1)	0.679	0.196
	HCV	2 (2.9)	12 (9.6)			3.8 (5.3)	8.8 (7.3)		
	Others	8 (11.4)	21 (16.8)			15.6 (21.8)	17.8 (14.6)		
AFP (%),ng/mL	<400	35 (50.0)	54 (43.2)	**0.444**	**0.137**	30.4 (42.6)	48.7 (40.0)	0.798	0.052
	≥400	35 (50.0)	71 (56.8)			41.0 (57.4)	72.9 (60.0)		
Nodules (%)	Single	24 (34.3)	43 (34.4)	1.000	0.002	24.2 (33.9)	39.9 (32.8)	0.906	0.024
	Multiple	46 (65.7)	82 (65.6)			47.2 (66.1)	81.7 (67.2)		
Tumor diameter, cm		81.0 (41.3,109.8)	108.0 (76.0,141.8)	**<0.001**	0.687	81.8 (47.8, 110.0)	108.2 (80.0,138.6)	0.002	0.681
PVTT (%)	No	28 (40.0)	8 (6.4)	**<0.001**	**0.964**	12.4 (17.3)	17.5 (14.4)	0.081	0.512
	Vp2	0 (0)	7 (5.6)			0 (0)	6.0 (4.9)		
	Vp3	22 (31.4)	72 (57.6)			27.6 (38.6)	66.0 (54.3)		
	Vp4	20 (28.6)	38 (30.4)			31.5 (44.1)	32.0 (26.3)		
Extrahepatic Spread (%)	No	28 (40.0)	105 (84.0)	**<0.001**	**1.017**	49.9 (69.9)	88.9 (73.1)	0.704	0.07
	YES	42 (60.0)	20 (16.0)			21.5 (30.1)	32.7 (26.9)		
ALBI (%)	1	28 (40.0)	41 (32.8)	0.469	0.182	30.9 (43.2)	37.5 (30.8)	0.043	0.512
	2	40 (57.1)	82 (65.6)			33.1 (46.3)	82.0 (67.5)		
	3	2 (2.9)	2 (1.6)			7.5 (10.5)	2.1 (1.7)		

Values in bold indicate statistically significant differences between groups (P < 0.05).

Before sIPTW, patients in quadruple therapy group underwent a median of 6 cycles of ICIs, a median of 4 months of anti-VEGF antibody/TKIs, and a median of 4 cycles of TACE-HAIC procedures, whereas patients in Targeted-Immunotherapy Group had a median of 7 cycles of ICIs and 4 months of anti-VEGF antibody/TKIs.

### 3.2 Efficacy

**Figure 1:**
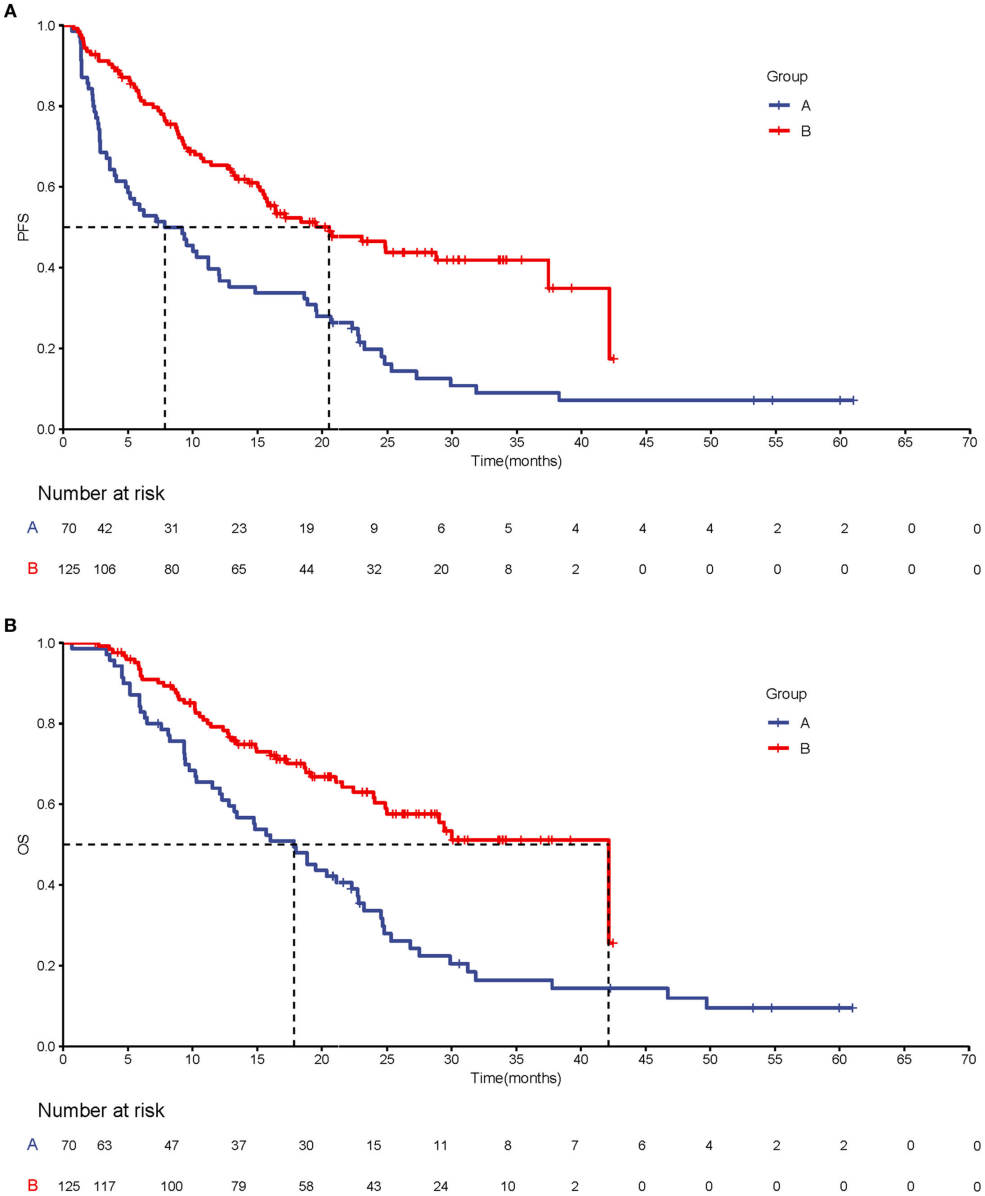
Kaplan–Meier analysis of progression-free survival **(A)** and overall survival **(B)** before sIPTW.

Prior to sIPTW, according to the mRECIST criteria, the quadruple therapy group exhibited a higher incidence of CR (22/125 vs. 0/70) and PR (66/125 vs. 14/70) compared to the targeted-immunotherapy group with the ORR (70.4% vs. 20%) and DCR (90.4% vs. 64.3%) also significantly greater in the former (**Table 2**). The quadruple therapy group demonstrated a significantly prolonged mPFS compared with the targeted-immunotherapy group (20.53 months (95% CI: [15.4-NA] vs. 7.83 months [95% CI: 4.80-12.8]; p<0.001; HR 95%CI 0.48 [0.32-0.72]), accompanied by superior PFS rates at both 12 and 24 months (65.4% vs.39.7% and 46.5% vs. 19.8%) (**Figure 1A**) (**Table 2**). Similarly, mOS was markedly improved in the quadruple therapy group relative to the targeted-immunotherapy cohort (42.1 months (95% CI: [25.0-NA] vs. 17.8 months [95% CI: 12.8-22.8]; p<0.001;HR 95%CI 0.53[0.34-0.85]),accompanied by superior OS rates at both 12 and 24 months(79.2% vs.64.0% and 61.7% vs. 33.6%). (**Figure 1B**) (**Table 2**)

**Table 2:** Tumor response.

Variables		Before IPTW			sIPTW		
		A(N = 70)	B(N = 125)	P	A(N = 71)	B(N = 122)	P
CR		0	22				
PR		14	66				
SD		31	25				
PD		25	12				
Objective response rate, n (%)		14 (20.0)	88 (70.4)	<0.001	20.0 (28.0)	83.1 (68.4)	0.001
Disease control rate, n (%),		45 (64.3)	113 (90.4)	<0.001	45.1 (63.1)	112.2 (92.3)	<0.001
PFS	months, median (95% CI)	7.83 (4.8–12.8)	20.53 (15.4–NA)	<0.001	10.0 (3.32–31.8)	16.4 (12.70–NA)	0.012
	HR(95% CI)	0.48(0.32–0.72)			0.48(0.31–0.75)		
	12–month PFS rate	39.7%	65.4%		42.1%	60.9%	
	24–month PFS rate	19.8%	46.5%		25.2%	42.9%	
OS	months, median (95% CI)	17.8 (12.8–22.8)	42.1 (25.0–NA)	<0.001	18.0 (14.7 –31.8)	29.4 (23.9– NA)	0.041
	HR(95% CI)	0.53(0.34–0.85)			0.53(0.34–0.85)		
	12–month OS rate	64.0%	79.2%		70.5%	74.4%	
	24–month OS rate	33.6%	61.7%		37.7%	59.6%	

**Figure 2:**
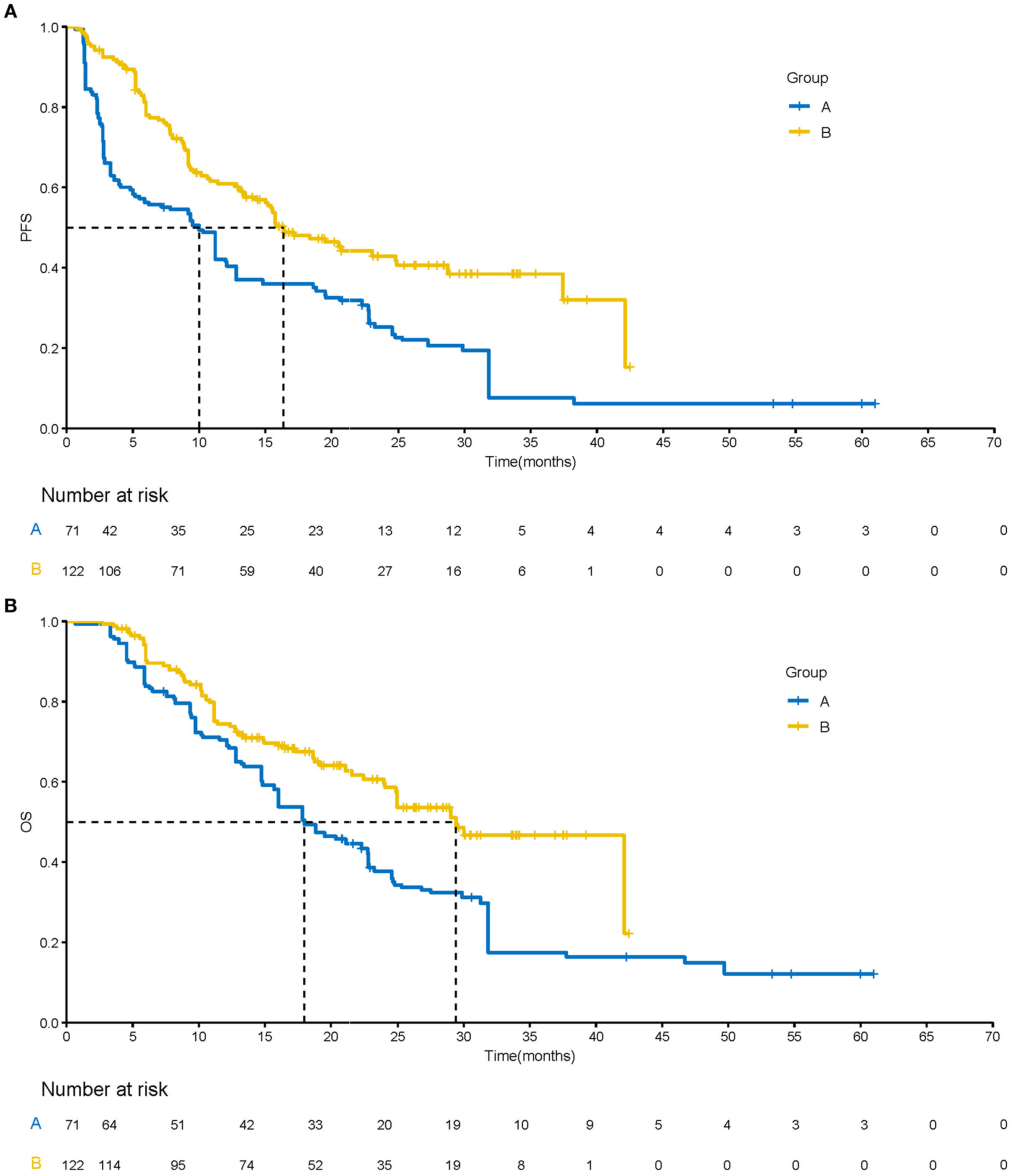
Kaplan–Meier analysis of progression-free survival **(A)** and overall survival **(B)** after sIPTW.

**Figure 3:**
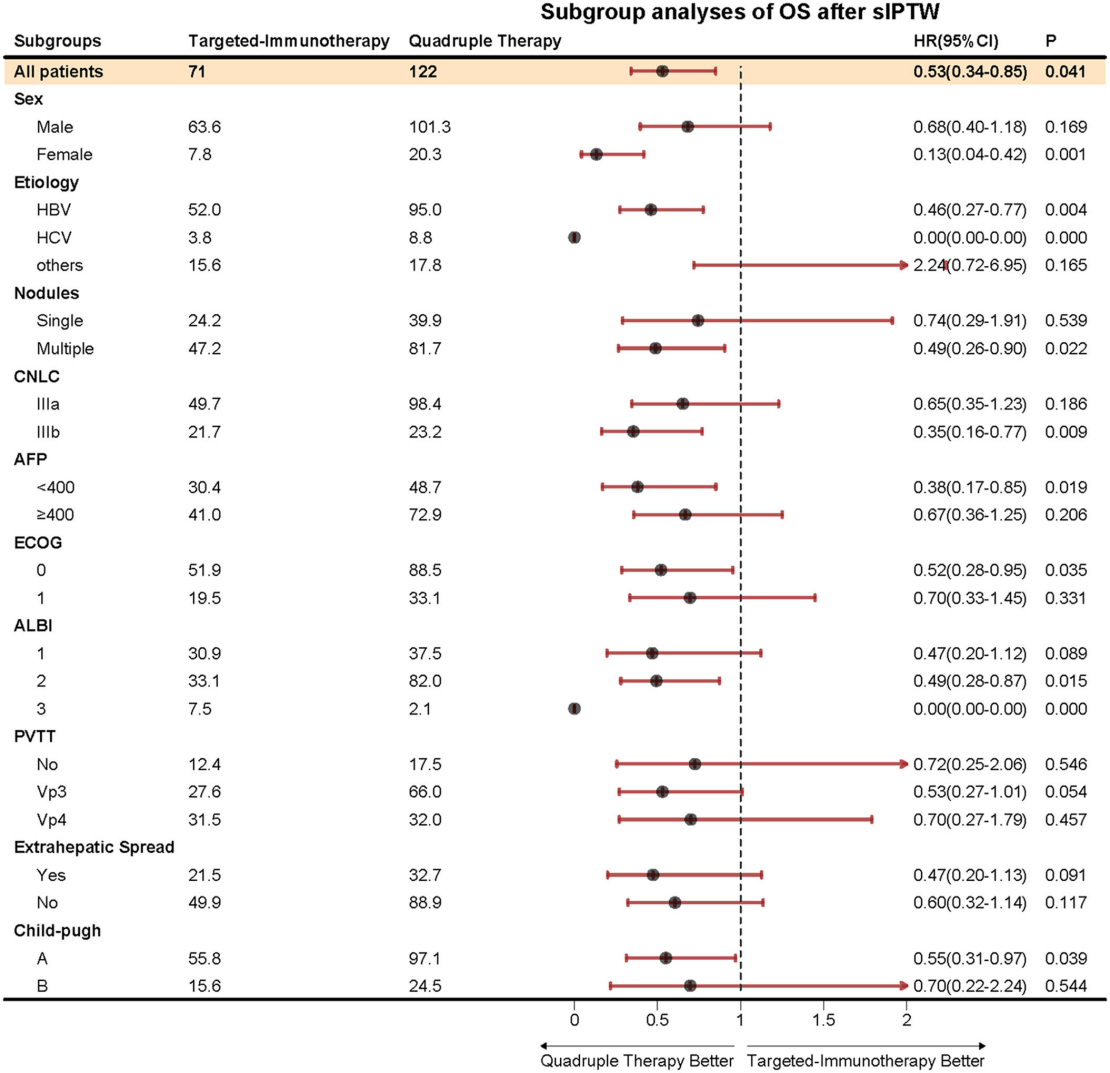
Subgroup analyses of OS after sIPTW.

After sIPTW, according to the mRECIST criteria, the quadruple therapy group exhibited a higher ORR and DCR compared to the targeted-immunotherapy group (68.4% vs. 28.0% and 92.3% vs. 63.1%) (**Table 2**). The quadruple therapy group demonstrated a significantly prolonged mPFS compared with the targeted-immunotherapy group (16.4 months (95% CI: [12.7-NA] vs. 10.0 months [95% CI: 3.32-31.8]; p<0.001; HR 95%CI 0.48 [0.31-0.75]), accompanied by superior PFS rates at both 12 and 24 months (60.9% vs.42.1% and 42.9% vs. 25.2%) (**Figure 2A**) (**Table 2**).Similarly, mOS was markedly improved in the quadruple therapy group relative to the targeted-immunotherapy group (29.4 months (95% CI: [23.9-NA] vs. 18.0 months [95% CI: 14.7-31.8]; p<0.001;HR 95%CI 0.53[0.34-0.85]),accompanied by superior OS rates at both 12 and 24 months(74.4% vs.70.5% and 59.6% vs. 37.7%). (**Figure 2B**) (**Table 2**). After sIPTW, Multivariate Cox regression analysis revealed that the receipt of quadruple therapy, the presence of multinodular hepatocellular carcinoma, and an ECOG performance status of 1 were independent prognostic factors influencing OS. (**Table 3**). In the subgroup analyses, the quadruple therapy group consistently demonstrated superior OS benefits across nearly all subpopulations when compared with the targeted-immunotherapy group (**Figure 3**).

**Table 3:** Univariable and multivariable analyses of OS after sIPTW.

Variables	Univariable			Multivariable		
	HR	95%CI	*P*	HR	95%CI	*P*
Group(B vs A)	0.57	0.34–0.95	0.030	0.53	0.34–0.85	0.008
Age	1.01	0.98–1.03	0.558			
Sex(male vs female)	1.42	0.78–2.59	0.253			
Etiology						
HCV vs HBV	0.59	0.22–1.63	0.310			
others vs HBV	0.77	0.44–1.36	0.371			
Nodules(single vs multiple)	0.55	0.32–0.95	0.031	0.54	0.31–0.93	0.026
PVTT type						
Vp2 vs no	0.66	0.28–1.56	0.346			
Vp3 vs no	0.86	0.46–1.62	0.644			
Vp4 vs no	0.82	0.41–1.63	0.563			
Extrahepatic spread (yes vs no)	1.11	0.67–1.85	0.693			
Child–pugh (B vs A)	0.98	0.63–1.51	0.918			
AFP≥400 (yes vs no)	1.52	0.92–2.51	0.104			
ALBI grade						
grade 2 vs grade 1	1.34	0.79–2.27	0.279			
grade 3 vs grade 1	0.96	0.57–1.60	0.872			
Tumor diameter, cm (≧̸10 vs <10)	1.16	0.73–1.83	0.531			
ECOG(1 vs 0)	1.90	1.18–3.08	0.008	1.84	1.13–3.00	0.014
CNLC(IIIb vs IIIa)	1.26	0.799–1.98	0.322			

### 3.3 Safety

**Figure 4:**
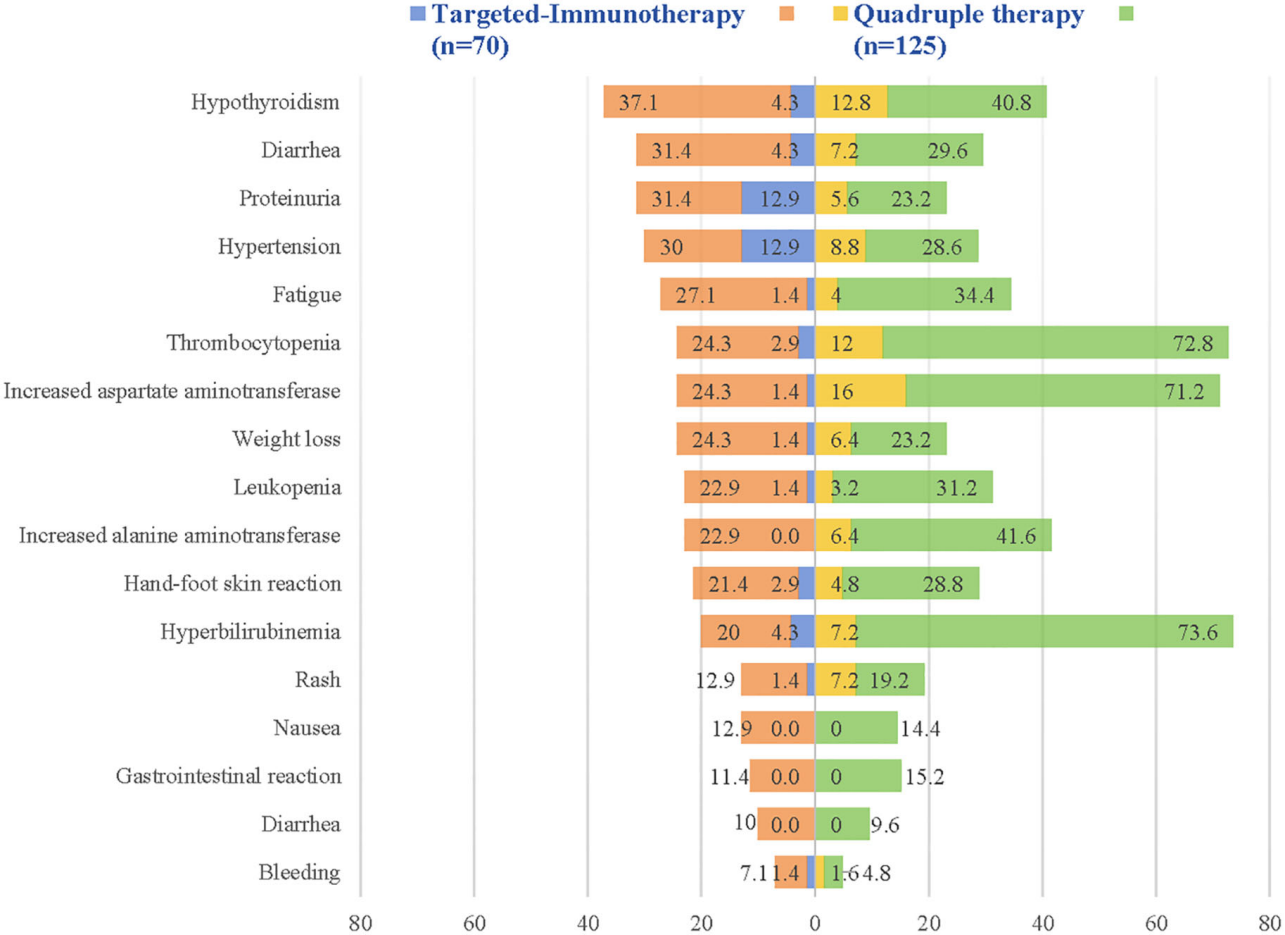
Major adverse events after treatment in whole population.

TRAE for both groups are detailed in supplement 1. Among the 119 patients in the quadruple therapy group and 68 patients in the targeted-immunotherapy group, AE of any grade occurred. Of these, 51 patients in the quadruple therapy group and 27 patients in the targeted-immunotherapy group experienced grade 3 or higher AE. Both groups reported one instance of grade 5 TRAE, which were attributed to sudden-onset hematemesis. Among the grade 3 or higher AE, the quadruple therapy group exhibited higher incidences of elevated total bilirubin (7.2% vs. 4.3%), thrombocytopenia (12.0% vs. 2.9%), increased alanine aminotransferase (ALT) (6.4% vs. 0%), aspartate aminotransferase (AST) (16% vs. 2.9%), diarrhea (7.2% vs. 4.3%), weight loss (6.4% vs. 1.4%), fatigue (4.0% vs.1.4%), and rash (7.2% vs. 1.4%), as well as hypothyroidism (12.8% vs. 4.3%), compared to the targeted-immunotherapy group (**Figure 4**).

## 4 Discussion

In this retrospective study, we assessed the efficacy and safety of a combined treatment regimen consisting of TACE, HAIC, Donafenib, and ICIs in comparison to the standard first-line therapy of targeted therapy plus ICIs for patients with BCLC stage C HCC. The results demonstrated that the addition of local therapies significantly improved treatment outcomes, including OS, PFS, ORR, and DCR compared to targeted therapy plus immunotherapy. Importantly, although the combination of local therapies was associated with a higher incidence of adverse events, no new safety signals emerged, and the existing adverse events were manageable.

This study primarily focuses on patients with BCLC stage C HCC, a population typically defined by substantial tumor burden, vascular invasion, or the presence of distant metastases. Several studies have indicated that HCC with PVTT is associated with notably poorer prognosis (20). In the present study, more than 80% of patients enrolled in both groups presented with PVTT; nevertheless, impressive outcomes were observed, including a median PFS of 16.4 months and a median OS of 29.4 months. These findings underscore that quadruple therapy represents a promising and effective therapeutic strategy for advanced HCC complicated by PVTT (10, 13, 21).

The combined efficacy of local and systemic treatments has gained widespread recognition (22), however, consensus on the integration of TACE and HAIC remains elusive. Some scholars argue that combining TACE and HAIC may significantly increase the hepatic burden, and that performing HAIC following TACE may compromise the efficacy of the infusion treatment (23). Conversely, other studies support the combined application of both therapies (12, 13). In this study, we similarly contend that combining TACE with HAIC can augment the efficacy of locoregional therapy. however, several practical considerations must be heeded during application. First, not every patient with advanced HCC necessitates the dual‐modality approach—particularly those with a modest intrahepatic tumor burden, for whom TACE or HAIC alone may prove adequate. Furthermore, during TACE, we aim to perform superselective catheterization of the tumor–feeding arteries to minimize damage to healthy liver tissue. For the main branches of the tumor–supplying arteries, we refrain from using particulate embolic agents, such as gelatin sponge or microspheres, for devascularization, ensuring that subsequent HAIC chemotherapy does not reflux into normal hepatic parenchyma. An additional rationale for combining TACE and HAIC lies in the observation that, in certain cases, the blood supply to hepatic tumors is not confined to the hepatic artery alone. It may also derive from the superior mesenteric artery, left gastric artery, or diaphragmatic arteries. In such instances, performing TACE on non–primary tumor–feeding arteries, while reserving HAIC for the main arterial supply, may offer a more effective strategy to capitalize on the advantages of local treatment (24).

In this study, we paid close attention to TRAEs. Compared to the targeted–immunotherapy group, the addition of TACE and HAIC resulted in a slight increase in overall adverse events (91.4% vs. 96.8%), particularly with regard to elevated ALT, AST, total bilirubin, decreased platelet count, as well as symptoms such as nausea, vomiting, and fever. However, the incidence of grade 3 or higher adverse events was similar between the two groups (38.6% vs. 40.8%). We observed that these AEs could be effectively managed by precise embolization, dynamically adjusting the chemotherapy drug infusion dose during HAIC, and promptly correcting relevant parameters and symptoms during the perioperative period, thereby reducing the occurrence or progression to grade 3 or higher. Furthermore, consistent with previous studies, no new safety concerns emerged in our study, which further supports the reliability and safety of the quadruple therapy treatment.

This study had several limitations. Firstly, it was a retrospective study, which inherently introduced a degree of selection bias in the patient enrollment process. Although we had employed Inverse Probability of Treatment Weighting (IPTW) to minimize baseline differences between the two groups, the retrospective nature still poses some challenges. As a real–world study, there was variability in the use of systemic therapeutic agents between the two groups, with dosing and frequency difficult to control, potentially impacting the accuracy of the results. Additionally, this is not a multicenter study, which limits the generalizability of the findings. Despite these limitations, the results of this study provide a valuable theoretical foundation for future prospective, multicenter investigations.

## 5 Conclusions

In conclusion, compared to targeted therapy combined with immunotherapy, the combination of TACE–HAIC with Donafenib and ICIs demonstrates superior outcomes in terms of PFS, OS, ORR, and DCR in patients with BCLC stage C HCC. Furthermore, the incidence of AE of grade 3 or higher was similar between the two groups. This study offers a novel therapeutic option for the treatment of BCLC stage C HCC.

## Data Availability

The original contributions presented in the study are included in the article/**Supplementary Material**. Further inquiries can be directed to the corresponding author.
