# Correction: TACE-HAlC combined with Donafenib and immune checkpoint inhibitors for BCLC stage C HCC patients (THEME study): a retrospective IPTW-adjusted cohort study

**DOI:** 10.3389/fimmu.2025.1745867

**Published:** 2025-11-20

**Authors:** Linan Yin, Weihang Li, Wencheng Shao, Xunbo Hou, Bowen Liu, Xuesong Liu, Ruibao Liu, Peng Huang

**Affiliations:** 1 Department of Interventional Radiology, Harbin Medical University Cancer Hospital, Harbin, China; 2 Department of Radiation Physics, Harbin Medical University Cancer Hospital, Harbin, China; 3 Department of Gastroenterology, Harbin Medical University Cancer Hospital, Harbin, China

**Keywords:** hepatocellular carcinoma, Donafenib, transarterial chemoembolization, hepatic arterial infusion chemotherapy, immune checkpoint inhibitors

Author “Peng Huang” was erroneously assigned as corresponding author. The correct corresponding author is “Peng Huang and Ruibao Liu”.

The original version of this article has been updated.

